# On the Mechanical Properties of *N*-Functionalised Dipeptide Gels

**DOI:** 10.3390/molecules24213855

**Published:** 2019-10-25

**Authors:** Ana M. Fuentes-Caparrós, Kate McAulay, Sarah E. Rogers, Robert M. Dalgliesh, Dave J. Adams

**Affiliations:** 1 School of Chemistry, University of Glasgow, Glasgow G12 8QQ, UK; 2315861F@student.gla.ac.uk (A.M.F.-C.); kate.mcaulay@glasgow.ac.uk (K.M.); 2 Rutherford Appleton Laboratory, ISIS Pulsed Neutron Source, Didcot OX11 0QX, UK; sarah.rogers@stfc.ac.uk (S.E.R.); robert.dalgliesh@stfc.ac.uk (R.M.D.)

**Keywords:** gel, hydrogel, dipeptide, rheology, SANS, fibre, network

## Abstract

The properties of a hydrogel are controlled by the underlying network that immobilizes the solvent. For gels formed by the self-assembly of a small molecule, it is common to show the primary fibres that entangle to form the network by microscopy, but it is difficult to access information about the network. One approach to understand the network is to examine the effect of the concentration on the rheological properties, such that G′∝ c^x^, where G′ is the storage modulus and c is the concentration. A number of reports link the exponent x to a specific type of network. Here, we discuss a small library of gels formed using functionalized dipeptides, and describe the underlying networks of these gels, using microscopy, small angle scattering and rheology. We show that apparently different networks can give very similar values of x.

## 1. Introduction

One effective method of preparing hydrogels is to use a functionalized dipeptide as a gelator [1,2,3,4,5]. Such gelators are able to form gels by self-assembling into a fibrous network that can immobilize the solvent. Gels can be formed in different ways, including pH switches or the in-situ formation of the dipeptide using an enzyme [2,3,6,7]. A highly effective method is to use a solvent switch approach, whereby the dipeptide is first dissolved in a water-miscible organic solvent, such as dimethyl sulfoxide (DMSO), and then water is added [8,9,10,11,12,13,14]. Gels form rapidly using this method, and their properties can be controlled by the ratio of solvent to water [11,15], the choice of solvent [10], as well as the concentration of the gelator that is used [8]. Gels formed using this approach have been shown to be useful for controlled release [8], cell culturing [13] as part of a composite, conducting gel [16] and 3D printing [17], for example [3,4].

Depending on the application, specific and different properties are required. For example, for controlled release, pore size is important, to control what can be trapped and the rate of release. Depending on the cells being cultured, specific rheological properties are usually required; for example, stem cell differentiation is known to be directed by the stiffness of the gels [18]. Hence, it is critical that the gel properties can be understood. These properties are controlled by the underlying fibre network. It is common to image the self-assembled fibres that entangle to form the network, for example, by transmission electron microscopy (TEM). However, the magnification used, the ability to observe only a tiny fraction of the sample, and the necessity of drying (conventional TEM) or the using thin films (cryo-TEM), means that it is difficult to infer much about the network. Small angle scattering can be used to access information on the solvated fibres, but, due to the accessible experimental range, limited information can be accessed about the network. Moving to ultra-small angle scattering opens up a wider size range, but there are limited data currently available [17,19], meaning it is again difficult to link the results to a specific network type. Confocal microscopy can be used to access a suitable length scale to observe the network, but there is normally limited 3D information available.

There have been a number of attempts to understand the underlying network of gels by plotting the storage modulus against concentration (c), such that G′ ∝ c^x^. A number of theories have been developed. For example, Macintosh theory predicts that a crosslinked network will result in a value of x of around 2.2 [20]. Values of x of around 4.5 are expected for colloidal gels [21,22], and an entangled semi-flexible network has been reported to give a value of x of 1.4 [23]. Values of x of around 2.5 have been reported for a peptide-based hydrogel [24], as well as for other low molecular weight gels [25]. Values of x of 2.4 and 4.6 were reported for two different peptide-based gels, the difference being a result of the peptide sequence [26]. The higher than expected value (assuming a semi-rigid network) of 4.6 was ascribed to fibre polydispersity and bundle formation, and, hence, the differences between the two systems are linked to whether a homogeneous or heterogeneous network was formed [26]. A value of 1.8 was reported for a di-Fmoc-lysine based gel [27]. Values of 1.8 and 3.7 have been reported for other peptide-based gels; the high value of 3.7 was again attributed to bundling [26]. There are a small number of examples where x has been calculated for functionalized dipeptide gels. We have reported values of around 1.4 for pH-triggered, dipeptide-based gels [28]. For solvent triggered gels formed from FmocFF, we found that x ranged from 1.3 to 1.8, depending on the solvent used, at a solvent:water ratio of 3:7 [10]. We also found that x depended upon the exact ratio of solvent to water, varying from 1.8 to 3 as the amount of co-solvent decreases, suggesting that differences in the networks could be correlated with x [10]. Elsewhere, it has been reported that FmocFF gels, formed at a range of ratios of DMSO:water, gave a value of x of 2.5 [15]. Hence, it is clear that a range of values have been reported, and there is an assumption that the value of x meaningfully describes the underlying network. However, it is still unclear how the value of x corresponds to the network, and even nominally identical systems have been reported to have different values [10,15].

Here, we describe gels formed of a library of functionalized dipeptides and discuss the underlying networks that lead to the gels. We show that these gels are formed of different types of network, with varying natures of underlying microstructure and domain size. Interestingly, gels that lead to a spherulitic microstructure present significant consistency in x values, although the domain sizes vary significantly. Moreover, we found that two apparently different underlying sizes of domain lead to the same x value.

## 2. Results and Discussion

**Figure 1:**
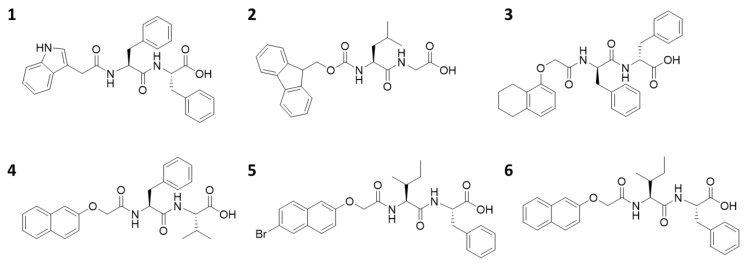
The chemical structures of the gelators used here, **1**–**6**.

**Figure 2:**
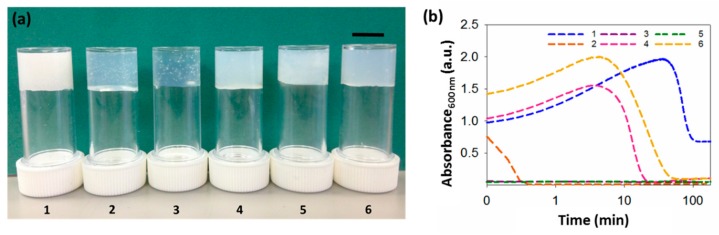
Gels and turbidity changes during gelation. (**a**) Photographs of gels formed from gelators **1**–**6.** The scale bar represents 1 cm. (**b**) Change in turbidity during gelation at 600 nm for gels **1**–**6**, showing different kinetics patterns. Logarithmic axis is used, due to time–scale similarities in the rate of assembly for some gels. All gels at a concentration of 5 mg mL^−1^ and a ratio of DMSO to water of 2:8.

Here, we use a small library of functionalized dipeptides as gelators (Figure 1). **1** was first reported by Martin et al. [29], and **2**–**6** come from our previously reported library [30]. In all cases, a dipeptide is conjugated to an aromatic moiety at the *N*-terminus. The *C*-terminus is free. To form the gels described here, the gelators are dissolved in dimethyl sulfoxide (DMSO), and then water is added, such that the final ratio of DMSO to water is 2:8. In all cases, a self-supporting material was formed (Figure 2a). We stress here the importance of ensuring that the gelation process is well controlled, to ensure high reproducibility of the final gels. A key parameter is ensuring that the temperature at which the gelation is carried out is carefully maintained. After gelation, the pH of the gels is between 3.7 and 4.5, which is typical for such samples [14,31], although not often stated.

In most cases, this process leads to an initially turbid solution as the water is added, followed by clarification over a period ranging from seconds to minutes (depending on the gelator) to form a relatively translucent gel (Figure 2a). This effect is common [9,11,15], and we have shown elsewhere that this is due to an initial phase separation, which is then followed by the formation of the fibrous network [11,31].

Whilst translucent or relatively translucent gels are formed when using **2**–**6**, gels formed using **1** are turbid. To better understand our systems, we examined the assembly kinetics, by measuring the changes in turbidity over time at 600 nm (Figure 2b). At this wavelength, the dipeptides do not absorb light, so changes here can be ascribed to changes in turbidity, arising from scattering. For gels formed using **1**, **4** or **6**, there is an initial increase in absorbance as soon as the water is added, corresponding with the nucleation phase, followed by a gradual decrease in turbidity; this can be ascribed to the formation of the fibres underpinning the gel phase, when the fibres start growing. For these three gels, the rate of change in turbidity is different, with the times at which the samples become less turbid being very different. The samples formed from **4** and **6** remain turbid for around 6 and 7 min, then become less turbid, with absorbance becoming essentially constant after 45 and 80 min, respectively. For **1**, turbidity persists for much longer (around 50 min) and the final gels are much more turbid, with no further changes after 120 min.

The gels formed using **2**, **3** or **5** show a different behavior. The initial turbidity persists for such a short period of time that there is no evidence of an increase in absorbance when the water is added, when following the changes using UV–Vis spectroscopy. Instead, absorbance decreases almost immediately after gelation begins. For gels formed using **2**, turbidity decreases within less than 1 min, after which absorbance remains constant and no further changes are observed. However, absorbance in samples formed from **3** and **5** remains constant almost immediately after gelation starts. Interestingly, gels **3** and **5** form smaller spherulitic domains, coinciding with the quicker rate of gelation. When the trigger is applied, phase separation takes place, followed by nucleation and growth. If the rate of gelation happens very quickly, the spherulitic structures will grow very fast, not allowing them to expand and, therefore, resulting in smaller spherulitic domains. In all cases, the kinetic profiles are similar at different concentrations (Figure S1, Supplementary Materials). Nevertheless, the higher the concentration, the more turbid the gels, resulting in a higher rate of absorbance.

**Figure 3:**
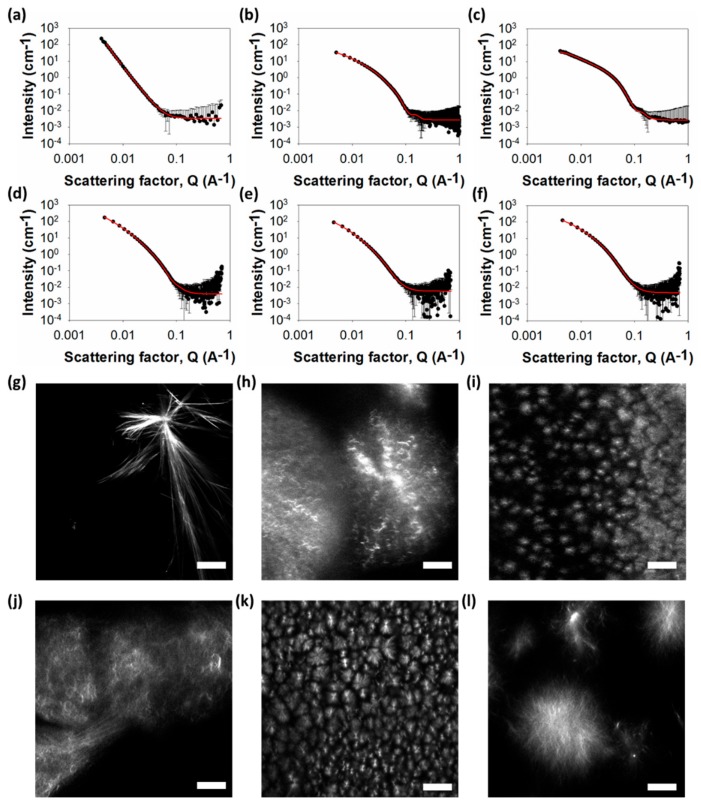
Small angle neutron scattering (SANS) and microscopy of gels. (Top) SANS scattering for (**a**)–(**f**) gel **1**–**6**. In all cases, the black circles represent the SANS data and the red lines show the fit to the data. (Bottom) Representative confocal microscopy images for gels formed from (**g**) **1**; (**h**) **2**; (**i**) **3**; (**j**) **4**; (**k**) **5**; (**l**) **6**. All gels at 5 mg mL^−1^ and the scale bar represents 20 µm in all cases.

To understand the underlying networks in these gels, we used small angle neutron scattering (SANS) and confocal microscopy. Confocal microscopy allows us to probe the microstructure without requiring us to dry the gels, which is known to lead to artefactual changes in the assembled structures [32]. Such issues are becoming more widely appreciated in the field [33]. Because of these drying issues, we have not used TEM or scanning electron microscopy (SEM) to probe the structures present in the gels. The primary structures underlying the network can also be probed without drying using SANS [34], with this information accessed via fits to the scattering data. The SANS data for the gel formed from **1** best fits to a power law (Figure 3a).

This implies that the structures underlying the network are large and outside the accessible Q-range over which the data were collected. In agreement with these assumptions, the microscopy shows the presence of long, rigid structures (Figure 3g). Polarized microscopy shows that these are crystalline (Figure S2, Supplementary Materials). Hence, for gels formed from **1**, it appears that the underlying structures are crystals, as opposed to fibres.

In comparison, the SANS data for gels formed from **2**–**6** best fit to a flexible elliptical cylinder model (Figure 3b–f), with radii in the range of 3–6 nm and axes ratios of 1.7–3. Confocal microscopy shows that, for all these gels, there is an underlying network of spherulitic domains (Figure 3; additional images are shown in Figures S3–S8, Supplementary Materials). For gels formed using this solvent-triggered approach, the addition of water causes a phase separation event, followed by nucleation and growth. This means that the underlying morphology of the fibres in these solvent-triggered gels is generally spherulitic [10,11]. The size of the spherulites varies between systems, with smaller spherulites forming in the gels that have arisen from **3** and **5**. Interestingly, this does not correlate with similar turbidity in bulk gel. Whilst the gel formed from **3** is the most translucent, that formed from **5** is relatively turbid. The gel formed using **2** is almost as translucent as that formed from **3**, although its spherulitic domains are much larger. This implies that there are different levels of solvation of fibres in each system. SANS data of each gel at different concentrations show no changes in scattering, confirming that no changes in the fibril structures happen when varying concentration (see Figure S9, Supplementary Materials).

The mechanical properties of these gels are typical of this class of dipeptide-based gel; the gels are frequency-independent and break at relatively low strain (see Figures S1 and S2, Supplementary Materials). The absolute values of the storage modulus (G′) and loss modulus (G″) depend on the gelator used, with the storage modulus varying from 1.000 Pa for **5**, to 200,000 Pa for **3**. Strain sweeps show that G′ is almost an order of magnitude greater than G″, indicating significant elastic behavior, characteristic of a gel. From tan δ (G″/G′, Figure S12), it is clear that gel **1** is different, compared to the rest of the gels. For gel **1**, tan δ increases after a strain of 0.1% is applied, indicative of the weakness of **1**. However, for gels **2**–**6**, tan δ remains constant up to 1% of strain, from which point an increase is observed, indicative of gel fracture, and a plateau stage, up to 100% of strain, at which point the gels break completely. Gels of **1**, using a solvent-triggered approach from DMSO, have been previously reported at this ratio of DMSO to water [29]. Data were provided for a concentration of 10 mg × mL^−1^, with a G′ of approximately 10,000 Pa. In our study, at the same concentration, G′ is around 30,000 Pa (Figure S13, Supplementary Materials), this being very similar to the value reported by Thordarson et al. [29].

Hence, from the above, it is clear that the gels are formed from different types of network, with varying types of underlying fibre microstructure and domain size. Despite this, the gels have relatively similar values of storage and loss modulus. To try to gain a deeper understanding of this, we examined the effect of concentration on the rheological data.

For the gels formed here, we measured the rheological data at different concentrations. The concentration range used had to be tailored to the gelator, as **1** has a higher minimum gelation concentration (mgc) than the others. The SANS data collected across the concentration ranges used for each of the gelators can all be fitted to the same model, with only slight changes in parameter. This shows that, in each case, the gels are the result of the same underlying structures. The data fitting for all concentrations are shown in Table S1, Supplementary Materials. Similarly, confocal microscopy shows that the microstructure of each case persists across the concentration range (Figures S14–S19, Supplementary Materials).

**Figure 4:**
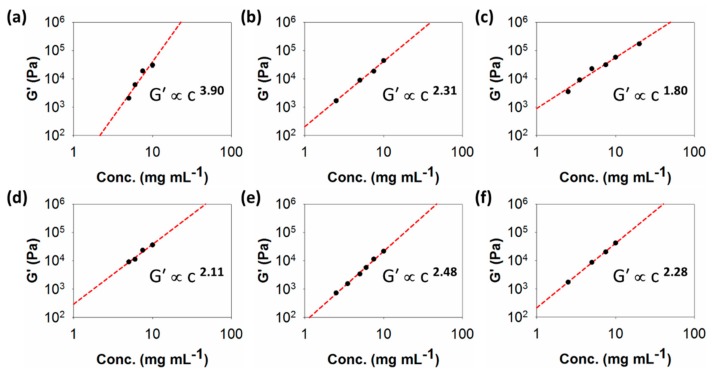
Plots of G′ against concentration for gels of (**a**) **1**; (**b**) **2**; (**c**) **3**; (**d**) **4**; (**e**) **5**; (**f**) **6**. The lines through the data show the fit used to determine the exponent x.

Plots showing G′ values against concentration for the different samples are shown in Figure 4. The values of G′ were taken from the plateau moduli in the linear viscoelastic region of the strain sweeps (Figures S13 and S20–S24). From these plots, the values of x are 3.90, 2.31, 1.80, 2.11, 2.48, and 2.28 for **1**–**6**, respectively. As such, there are significant differences between the values of x for these gels. We highlight gel **1** as having the highest value of x.

The models developed linking G′ and concentration typically report exponents of around 1.4 for an entangled semi-flexible network [23]. Such a value has been reported for pH-triggered dipeptide gels [28], which have a very different, more uniform fibre microstructure [35]. Values of around 2.2 are typical of cross-linked biopolymer networks [20]. The majority of the values here are around this value, but it is clear that the gels are formed of spherulitic domains of varying size. The value of 3.90 for **1** falls within the expected range expected colloidal gels.

Another peptide-based gel that has been found to have an exponent of 2.48 is MAX-1 [36]. Direct comparison with **5**, which has the same exponent, is therefore interesting. Whilst fracture into large domains has been suggested under shear [37], initially, such gels are assumed to be relatively uniform in fibre distribution [19,36], unlike the clear spherulitic domains seen for **5**. Hence, it seems that the exponent alone cannot easily be used to imply a certain type of network.

Another model is that of Jones et al. [38], which links the exponent to a fractal dimension such that:

G′ = c^(3 + D^_F_^)/(3 − D^_F_^)^ (1)

where D_F_ is the fractal dimension of the interconnecting objects, where the objects forming the network are straight, and, hence, have a fractal dimension of 1, G′ ∝ c^2^, i.e., x = 2. This has been shown to be the case for some peptide-based gels [26], as well as gels formed by a modified amino acid [27]. This is broadly true for **2**–**6**, here. However, as pointed out elsewhere [26], the model does not take polydispersity into account, hence the differences in fibre stiffness. This explanation has been used to account for the higher values of x [26]. It may be that this explanation holds for gels formed by **1**, where the objects imaged by confocal microscopy are clearly straight but are clearly laterally aggregated in some cases.

## 3. Materials and Methods

### 3.1. Materials

Compounds **2**–**6** were synthesized following previously described methods [28,30]. DMSO was purchased from Fisher Scientific (Pittsburgh, PA, USA) and DMSO-d_6_ and D_2_O were acquired from Sigma Aldrich (St. Louis, MO, USA). All other starting chemicals were purchased from Sigma Aldrich.

Compound **1** was prepared as follows: 3-Indole acetic acid (1.32 g, 0.00753 mol) was suspended in chloroform (50 mL) and N-methylmorpholine (0.83 mL, 0.00753 mol) was added. The solution was cooled in an ice bath. In a separate flask, the trifluoroacetate salt of diphenylalanine ethyl ester [39] (3.42 g, 0.00753 mol) was suspended in chloroform (30 mL), and N-methylmorpholine (0.83 mL, 0.00753 mol) added. To the first flask was added isobutylchloroformate (0.98 mL, 0.00753 mol). After approximately 3 min, the second solution was added, and the flask washed with chloroform (20 mL), into the solution. The mixture was stirred overnight, before being washed with water, and then acidic water, followed by water again. The solution was dried using magnesium sulfate and dried in vacuo. The crude mixture was used in the next step directly.

The crude mixture was dissolved in THF (30 mL), and water added (10 mL). To this, lithium hydroxide (0.3 g) was added and the mixture stirred. Periodically, small aliquots were removed and added to an excess of water. When no precipitate was observed, water was added to the mixture (200 mL), and the solution acidified to pH 3. The resulting precipitate was collected by filtration and dried, to give **1** as pox an off-white solid (2.37 g, 67% over the two steps).

δ_H_ (400 MHz, DMSO-d_6_) 12.77 (1H, br s, COOH), 10.79 (1H, s, indole-NH), 8.30 (1H, d, *J* 7.76, NH), 7.97 (1H, d, *J* 8.44, NH), 7.32–7.17 (12H, m, H_Ar_), 7.05–6.99 (2H, m, H_Ar_), 6.91–6.87 (1H, m, H_Ar_), 4.58–4.53 (1H, m, CH^*^), 4.46–4.41 (1H, m, CH^*^), 3.47 (1H, d, *J* 15.32, indole-CH_a_H_b_), 3.42 (1H, d, *J* 15.27, indole-CH_a_H_b_), 3.05 (1H, dd, *J* 13.90, 5.33, PhCH_2_), 2.97 (1H, dd, *J* 13.78, 4.05, PhCH_2_), 2.91 (1H, dd, *J* 13.93, 8.54, PhCH_2_), 2.72 (1H, dd, *J* 13.75, 9.80, PhCH_2_). *δ*_C_ (100 MHz, DMSO-d_6_) 172.72, 171.30, and 170.44 (C=O), 137.71, 137.35, 136.05, 129.27, 129.13, 128.21, 127.92, 127.19, 126.47, 126.17, 123.76, 120.87, 118.66, 118.28, 111.18, and 108.57 (C_Ar_), 53.51 and 53.45 (CH^*^), 37.52 and 36.71 (PhCH_2_), 32.33 (indole-CH_2_). HRMS (ESI) *m*/*z*: [M + Na]^+^ calcd for C_28_H_27_N_3_NaO_4_ 492.1894; found 492.1911.

### 3.2. Sample Preparation

Stock solutions of gels **1**–**6** were prepared at different concentrations by dissolving the pre-weighed amount of the required gelator in dimethyl sulfoxide (DMSO), such as the final ratio of DMSO:water was 2:8. Deionised water was added in one aliquot to each solution, to make up a final volume of 2 mL, and the samples were left overnight at room temperature, without being disturbed, to allow gelation to occur. After gelation, the pH of the gels was between 3.7 and 4.5.

For the small angle neutron scattering experiments, deuterated DMSO (DMSO-d_6_) and D_2_O were used, instead of DMSO and water.

### 3.3. pH Measurements

A calibrated FC2020 pH probe (Hanna Instruments, Bedford, UK) was used for pH measurements. The stated accuracy of pH measurements is ±0.1.

### 3.4. Oscillatory Shear Rheology

All rheological measurements were undertaken on an MCR 101 or MCR 301 rheometer (Anton Paar, Graz, Austria) using the software Rheoplus/32 v3.40. A cup and vane geometry (Anton Paar, Graz, Austria) was used throughout, to perform strain and frequency sweeps. In all cases, 2 mL of the gels were prepared in 7 mL Sterilin vials (Fisher scientific, Leicestershire, UK) and left to stand overnight at room temperature, prior to the measurements. Strain sweeps were performed at 10 rad × s^−1^ from 0.01% to 1000% strain. Frequency sweeps were collected at a range of frequencies varying between 1 rad × s^−1^ to 100 rad × s^−1^, at 0.5% strain, to guarantee the measurements were being carried out in the linear viscoelastic region. All measurements were performed at 25 °C.

### 3.5. Confocal Microscopy

A Zeiss LSM710 confocal microscope (Zeiss, Gottingen, Germany) and software Carl Zeiss ZEN 2011 v7.0.3.286 with an LD EC Epiplan NEUFLUAR 50X, 0.55 DIC (Carl Zeiss, White Plains, NY, USA) objective was used. All samples were prepared in a CELLviewTM (Greiner Bio-One, Stonehouse, UK) 35 mm plastic cell culture dish with a borosilicate glass bottom. The samples were stained with 2 µL × mL^−1^ of a 0.1 *w*% Nile Blue A solution, and excited at 634 nm using a He–Ne laser (Zeiss, Gottingen, Germany). All samples were prepared in situ as described previously, at a volume of 400 µL, and left overnight to gel.

### 3.6. UV–Vis Measurements

Absorbance spectra for gels **1**–**6** were collected over time (120 min) at 600 nm on an Agilent Cary 60 UV–Vis spectrophotometer (Agilent Technology, Selangor, Malaysia) using the software Cary WinUV, kinetic Application v5.0.0.999. All samples were prepared in a 1 mm path length quartz cuvette. Firstly, water was added into the cuvette and then a DMSO solution containing the gelator was added, such that the final ratio of DMSO:water was 2:8. After DMSO was added, the mixture was mixed quickly with the help a needle, as some of the gelators start to gel after few seconds. The final volume of gel examined was 300 µL, at a range of different concentrations, for gels **1**–**6** at 25 °C.

### 3.7. Small Angle Neutron Scattering (SANS)

Gels were prepared as described above, in UV spectrophotometer quartz cuvettes with 2 mm path length. All measurements were performed at 25 °C, using the Larmor instrument (ISIS, Rutherford Appleton Laboratory, Didcot, UK), using a wavelength band of 0.9 to 13 Ang to access a q range of 0.004 to 0.7 Ang^−1^. Data was reduced and corrected using the Mantid framework [40]. Fittings were carried out using the SasView software [41].

## 4. Conclusions

In conclusion, we have described gels formed from a small library of functionalized dipeptides. Exponents and fractal dimensions calculated from the rheological data are consistent with other peptide-based gels and, also, with these, being generally densely cross-linked gels. Using this solvent-triggered approach results in spherulitic domains of fibres in most cases, although one gel contains crystalline structures. These are not consistent with the typical cartoon of a densely cross-linked, peptide-based gel. We also found that gels with very similar sizes of spherulitic domain have different exponents from G′ ∝ c^x^. This all highlights that using exponents from rheological data to infer information about the networks should be done with caution.

## Supplementary Materials

The following are available online at https://www.mdpi.com/1420-3049/24/21/3855/s1, further rheology and confocal microscopy images.
