# Supplementary material for: On the Mechanical Properties of N-Functionalised Dipeptide Gels
Source: Molecules. 2019 Oct 25;24(21):3855. doi: 10.3390/molecules24213855 (PMC6864704; doi:10.3390/molecules24213855)
Supplement: Supplementary file 1 [file molecules-24-03855-s001.pdf]

On the Mechanical Properties of N-Functionalised Dipeptide Gels

Ana M. Fuentes-Caparrós,[a] Kate McAulay,[a] Sarah Rogers,[b] Robert Dalgliesh [b] and Dave J. Adams*[a]

a School of Chemistry, University of Glasgow, Glasgow, G12 8QQ, U.K.
b ISIS Pulsed Neutron Source, Rutherford Appleton Laboratory, Didcot, OX11 0QX, U.K.

SUPPORTING INFORMATION

Supplementary Figures

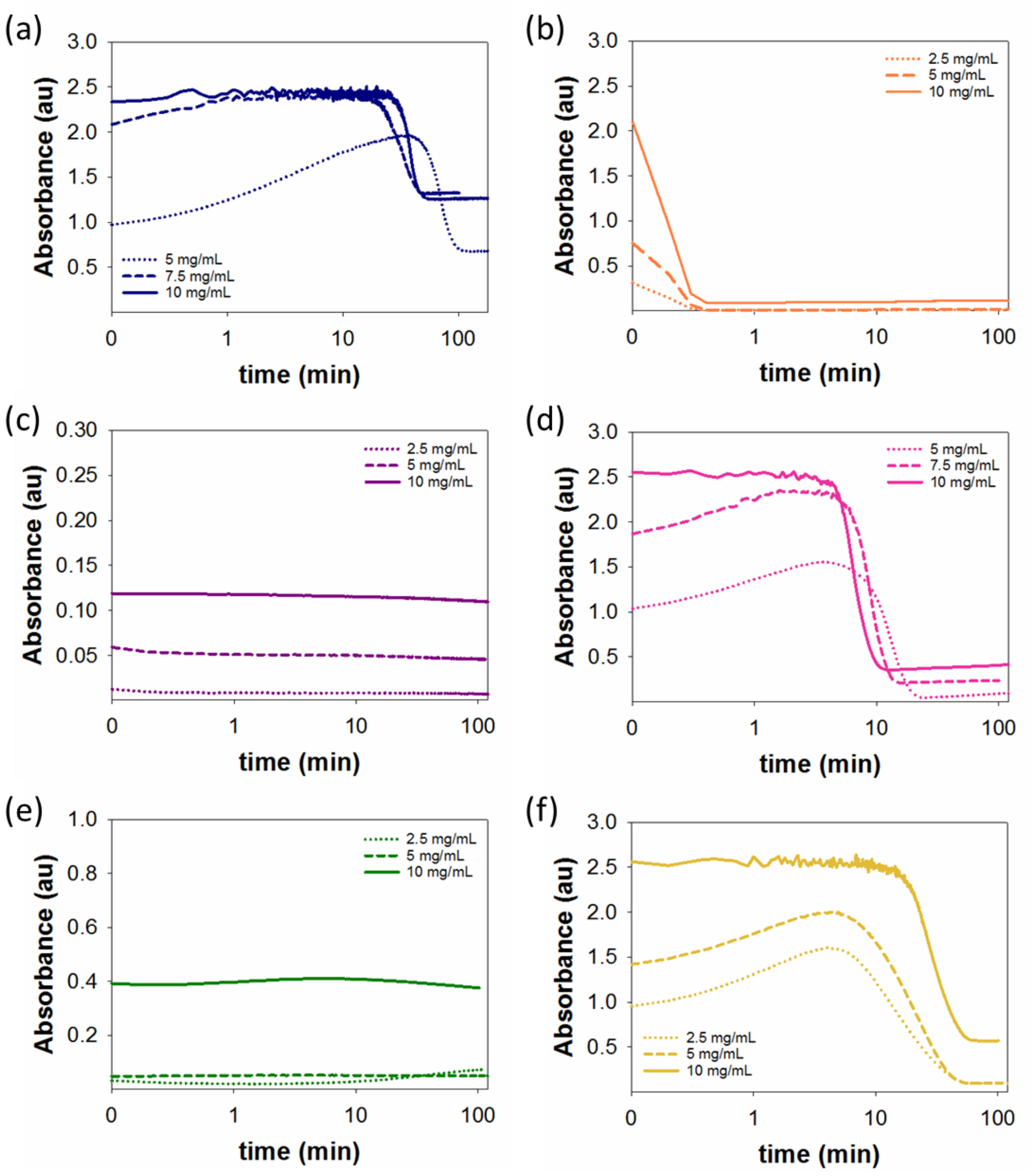

Figure S1. Changes in turbidity during gelation at 600 nm for gels (a) **1**; (b) **2**; (c) **3**; (d) **4**; (e) **5** and (f) **6** at a range of different concentrations. All gels at a ratio of DMSO to water of 2:8. For gels **1**, **4** and **6** the absorbance at the higher concentrations top out since they are very turbid. In all cases, the kinetic profile is the same at different concentrations. As the concentration is increased, the gel is more turbid, therefore, resulting in a higher absorbance. For gel **5,** absorbance at 2.5 and 5 mg mL^{-1} is very similar. We attribute this similarity as a result of different solvent/water mixing in different systems as the gelation happen in a matter of seconds.

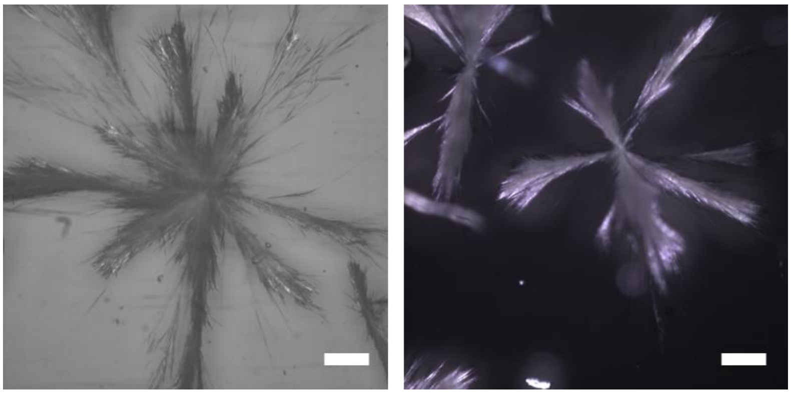

Figure S2. Polarised microscopy images showing the crystallinity of the structures within the gel of **1**. The scale bar represents 200 μm

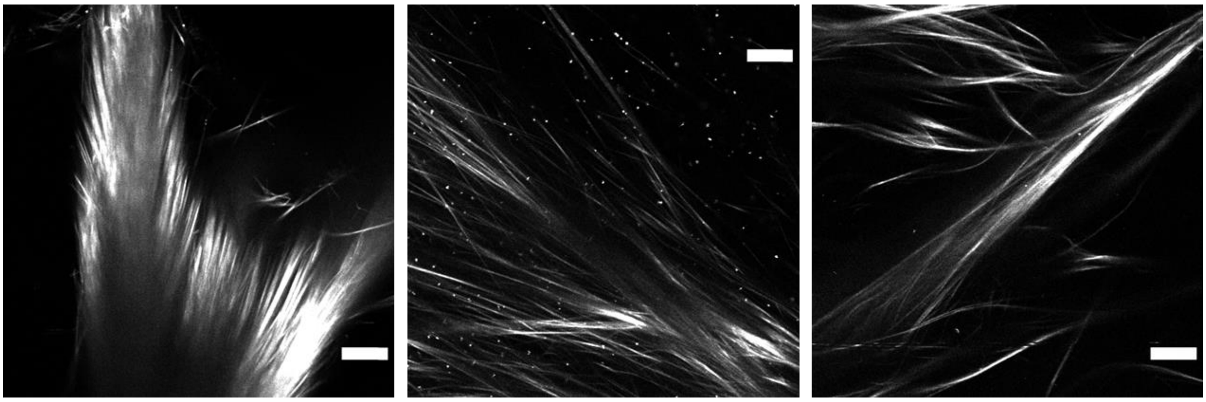

Figure S3. Further confocal images for gels of **1** prepared at a concentration of 5 mg·mL^{-1} and a DMSO:water ratio of 2:8. The scale bar represents 20 μm.

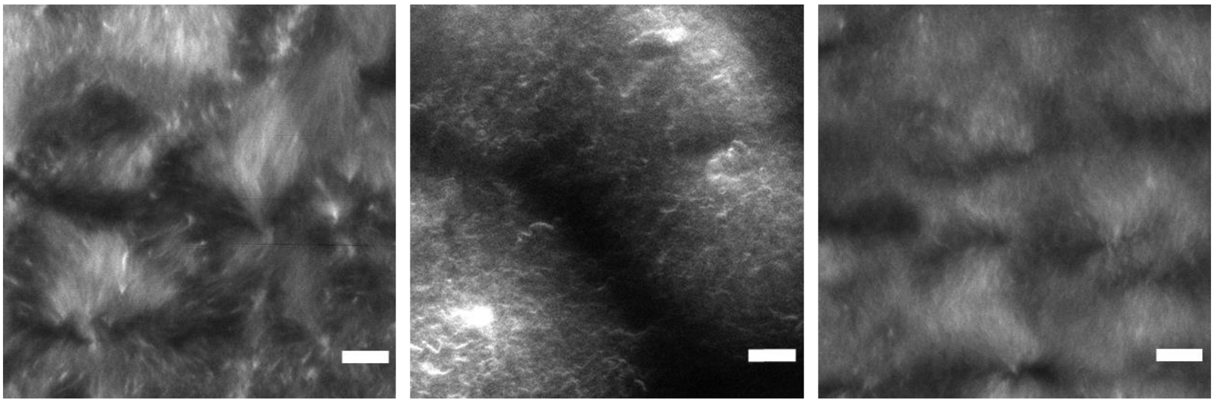

Figure S4. Further confocal images for gels of **2** prepared at a concentration of 5 mg·mL^{-1} and a DMSO:water ratio of 2:8. The scale bar represents 20 μm.

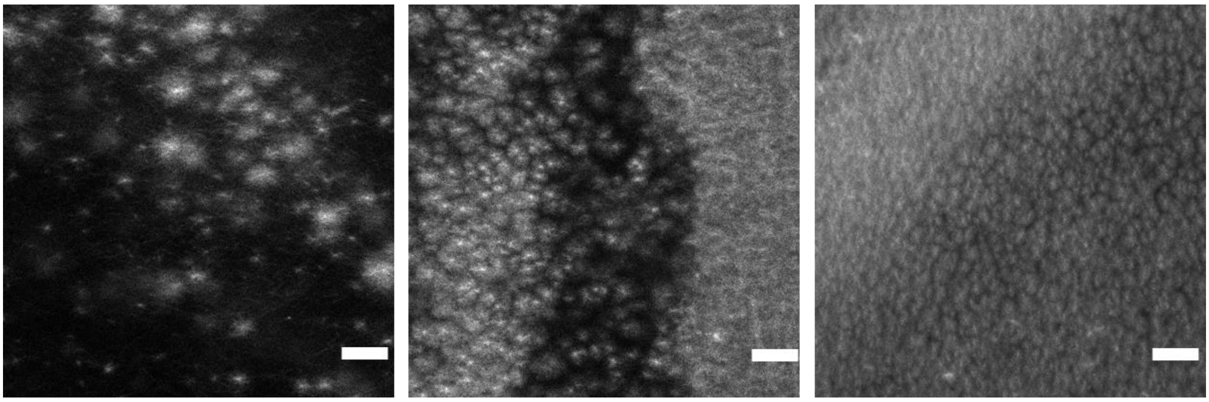

Figure S5. Further confocal images for gels of **3** prepared at a concentration of 5 mg·mL^{-1} and a DMSO:water ratio of 2:8. The scale bar represents 20 μm.

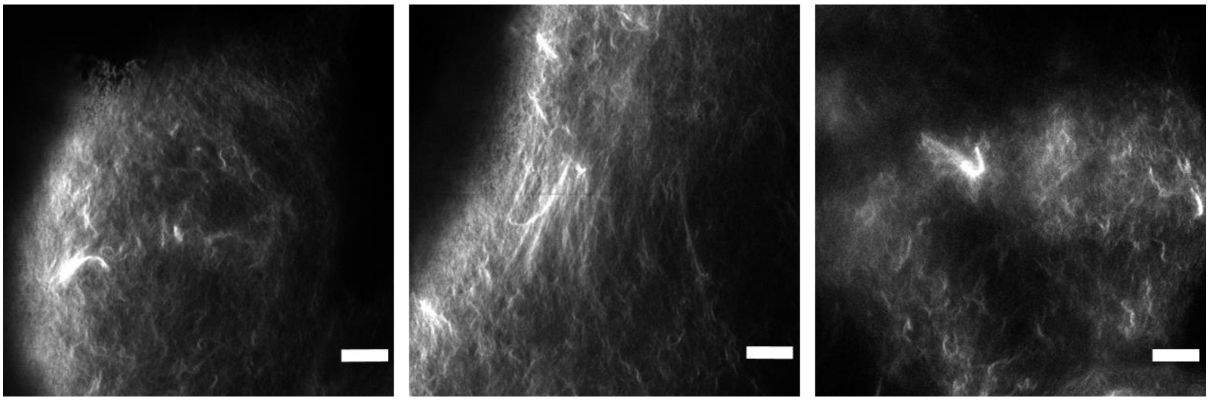

Figure S6. Further confocal images for gels of **4** prepared at 5 mg/mL and a DMSO:water ratio of 2:8. The scale bar represents 20 μm.

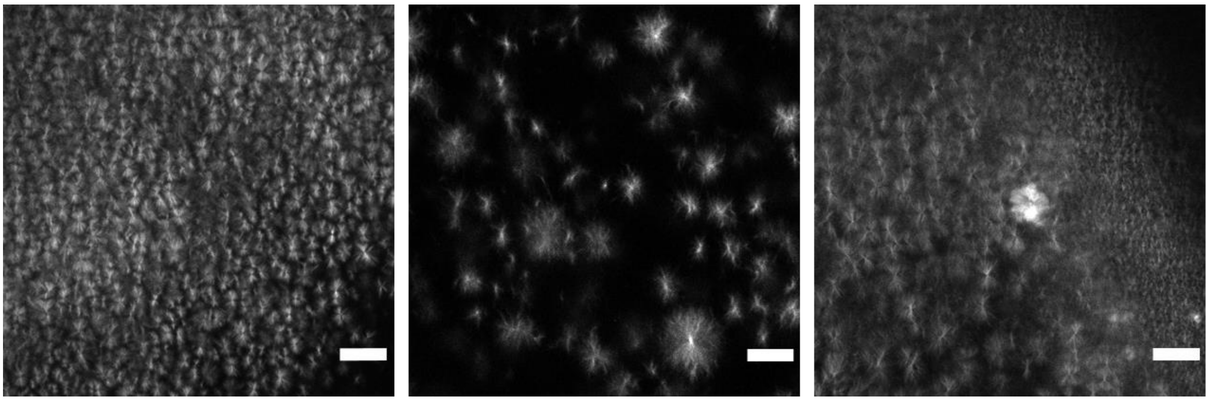

Figure S7. Further confocal images for gels of **5** prepared at a concentration of 5 mg·mL^{-1} and a DMSO:water ratio of 2:8. The scale bar represents 20 μm.

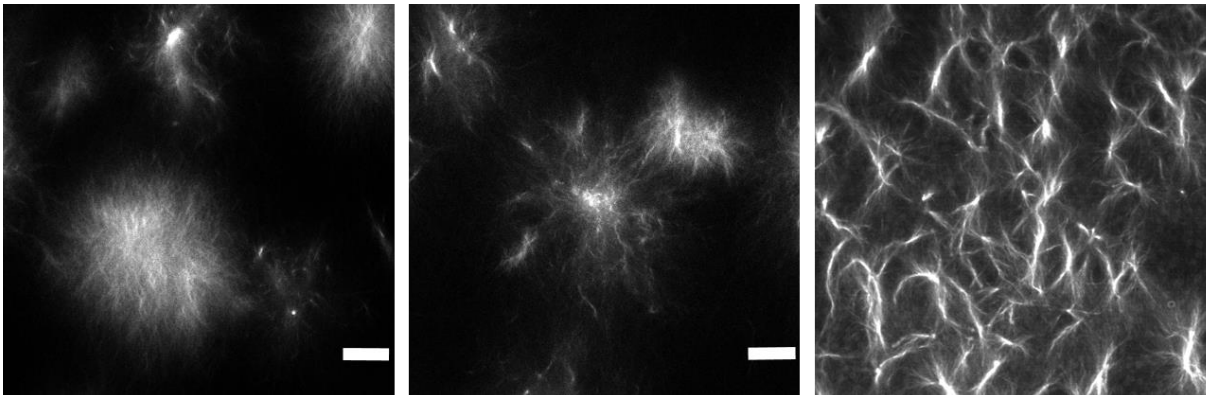

Figure S8. Further confocal images for gels of **6** prepared at a concentration of 5 mg·mL^{-1} and a DMSO:water ratio of 2:8. The scale bar represents 20 μm.

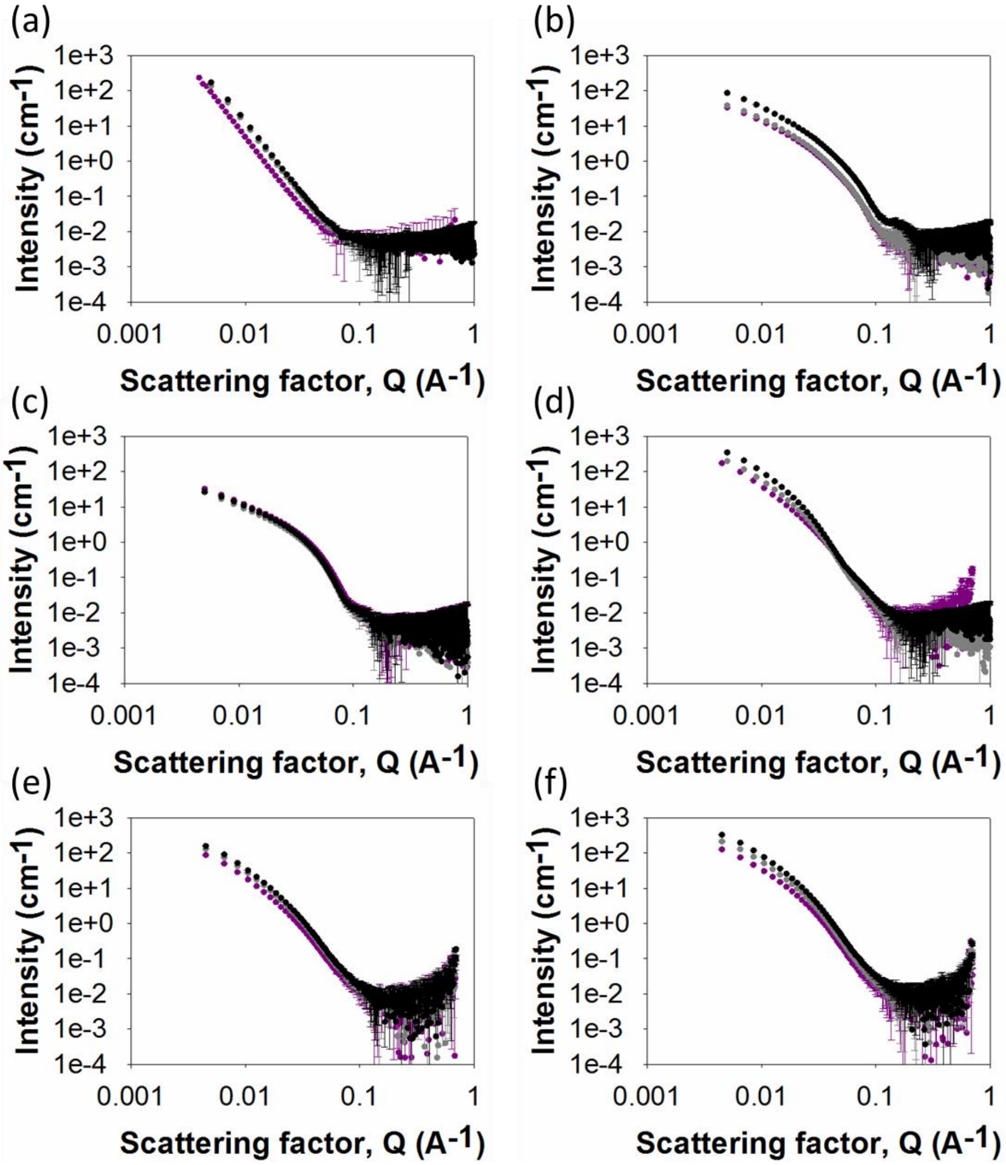

Figure S9. SANS scattering for (a)-(f) gel **1-6** at 5 mg mL^{-1} (purple data), 7.5 mg mL^{-1} (grey data) and 10 mg mL^{-1} (black data).

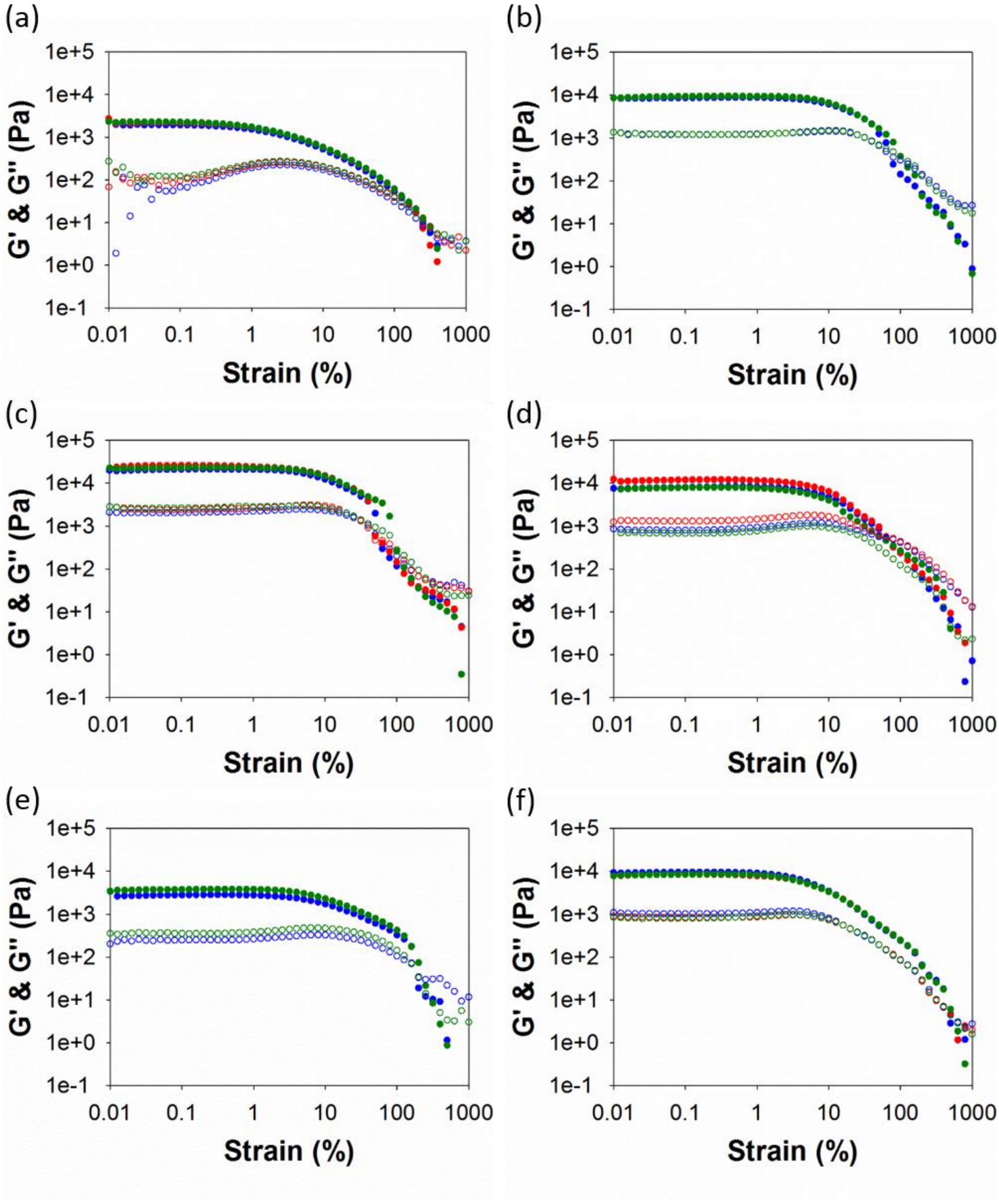

Figure S10. Strain sweeps for gels prepared at a concentration 5 mg·mL^{-1} and a DMSO:water ratio of 2:8. Data are shown for gels of (a) **1**; (b) **2**; (c) **3**; (d) **4**; (e) **5**; (f) **6**. In each case, separate datasets are provided for three nominally identical gels; for (b), three datasets are present, but the values are so close that the third set is hidden.

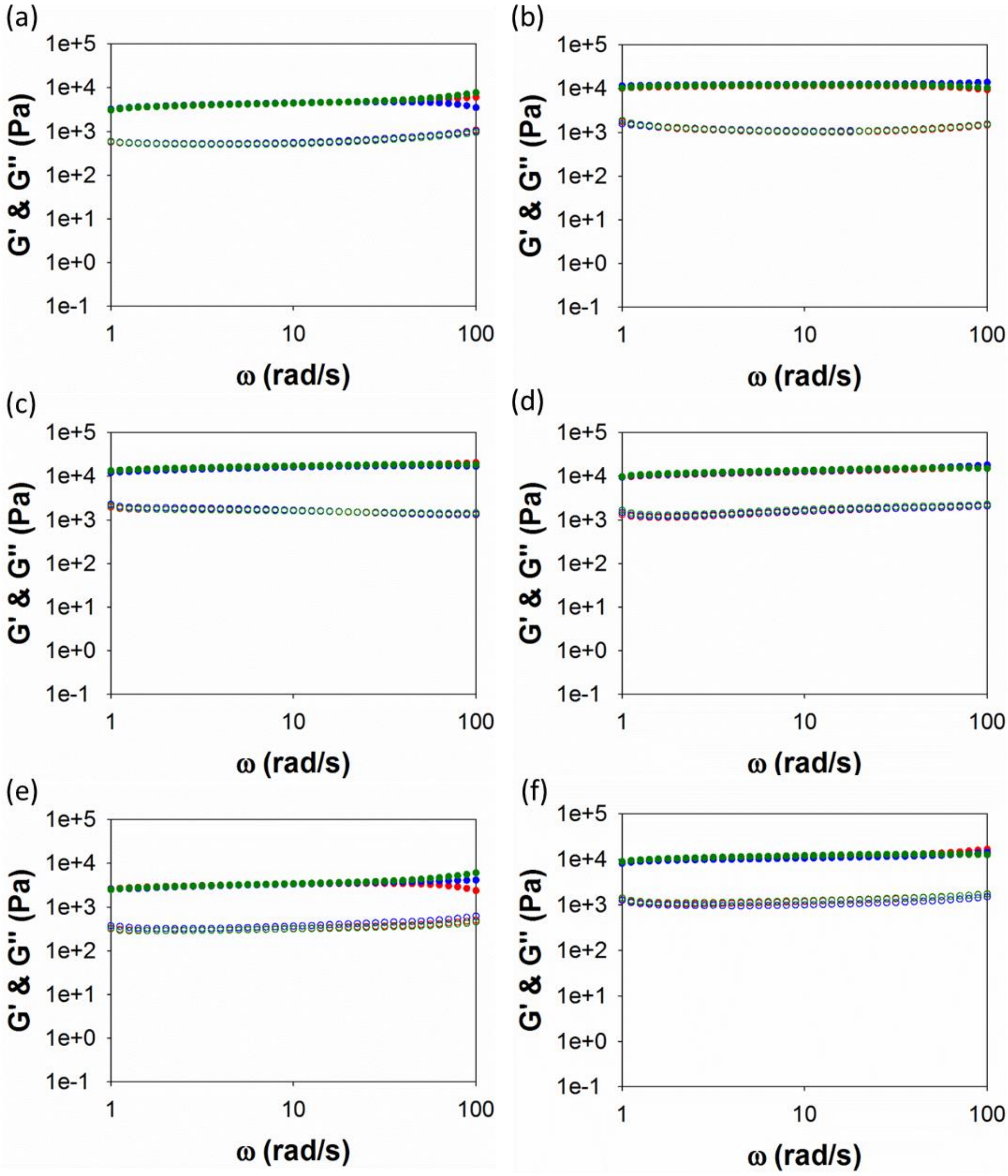

Figure S11. Frequency sweeps for gels prepared at a concentration of 5 mg·mL^{-1} and a DMSO:water ratio of 2:8. Data are shown for gels of (a) **1**; (b) **2**; (c) **3**; (d) **4**; (e) **5**; (f) **6**. In each case, separate datasets are provided for three nominally identical gels.

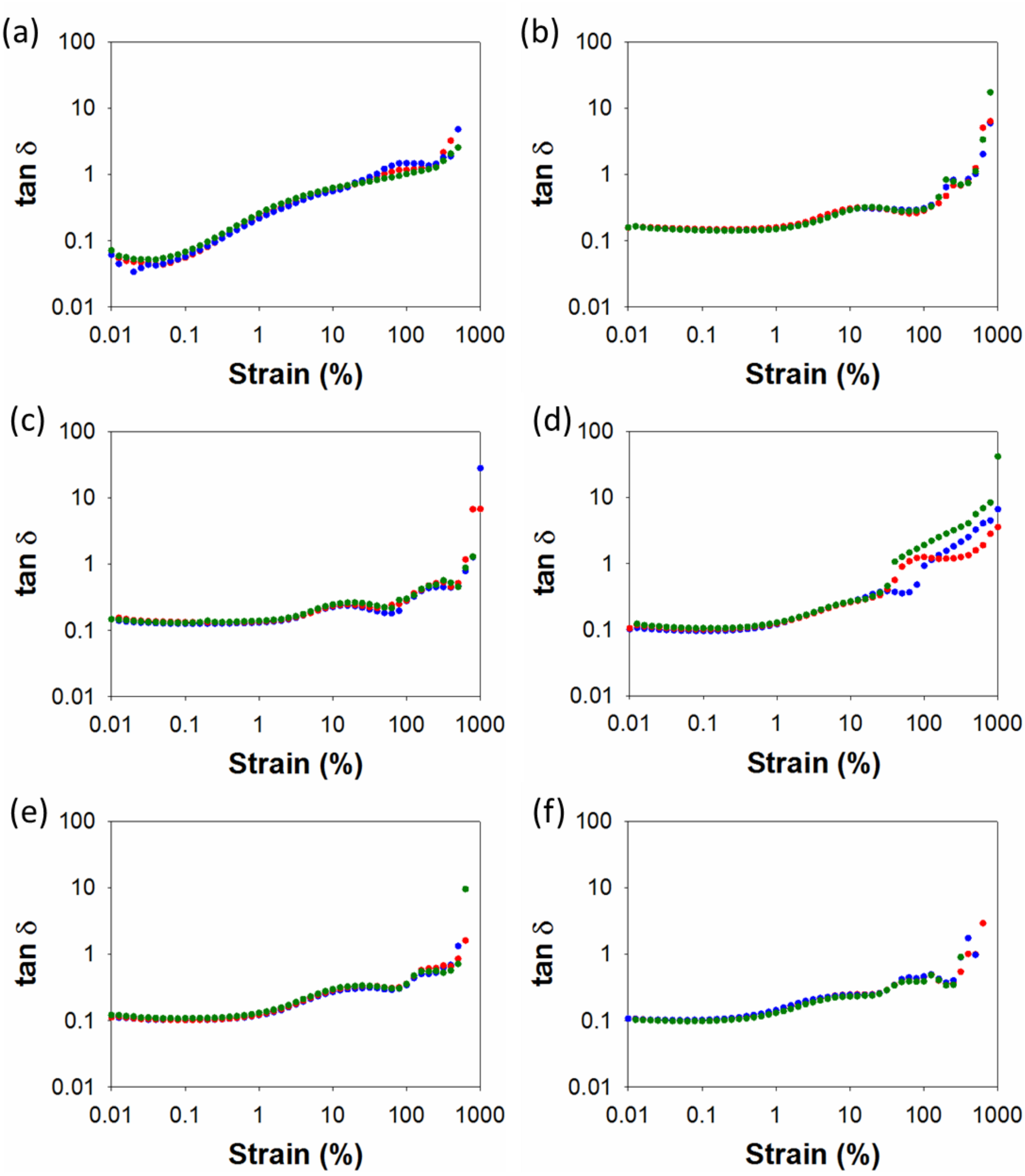

Figure S12. Tan delta (G''/G') against strain for gels of (a) **1**; (b) **2**; (c) **3**; (d) **4**; (e) **5**; (f) **6** prepared at 10 mg mL^{-1} at a DMSO:water ratio of 2:8.

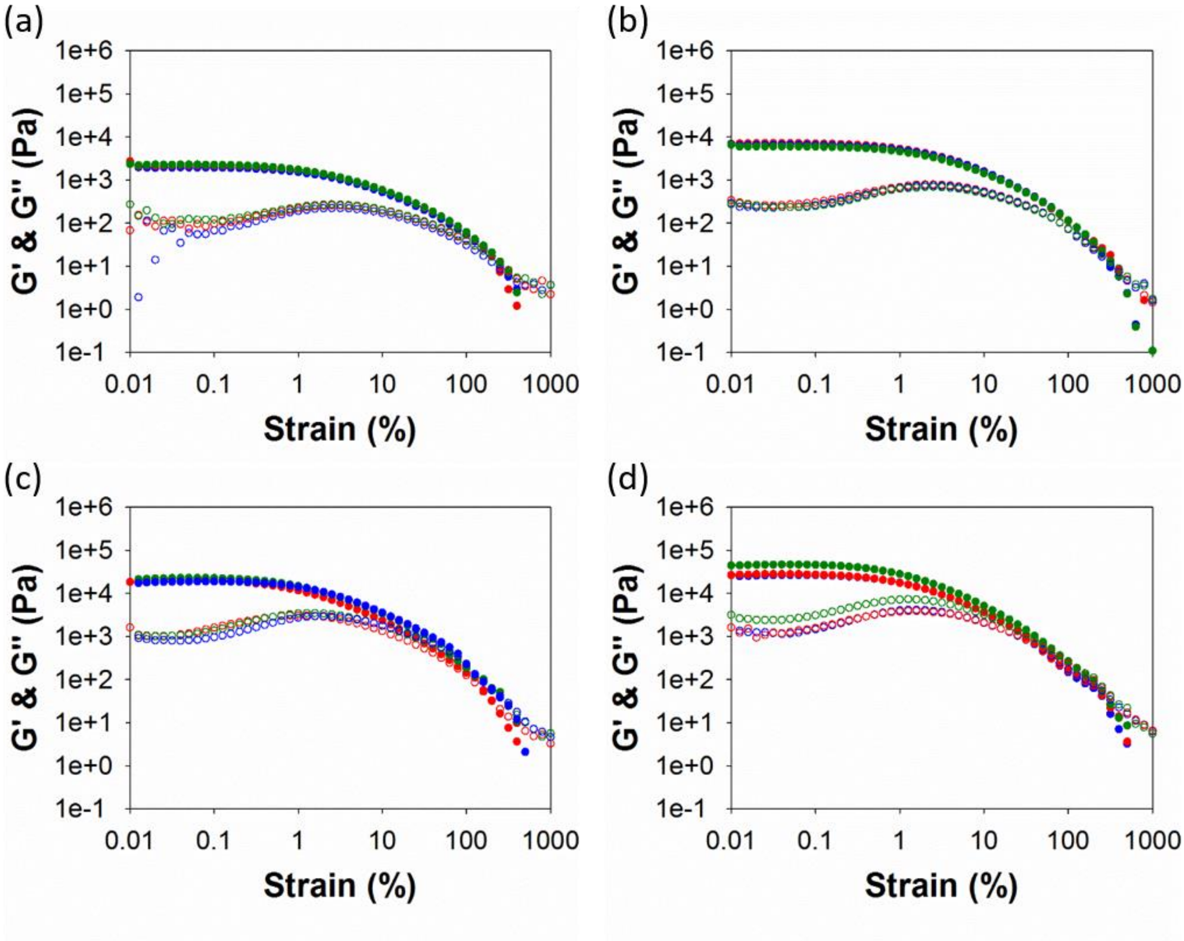

Figure S13. Strain sweeps for gels of **1** prepared at different concentrations at a DMSO:water ratio of 2:8. Concentrations are (a) 5 mg·mL^{-1}; (b) 6 mg·mL^{-1}; (c) 7.5 mg·mL^{-1}; (d) 10 mg·mL^{-1}. In each case, separate datasets are provided for three nominally identical gels.

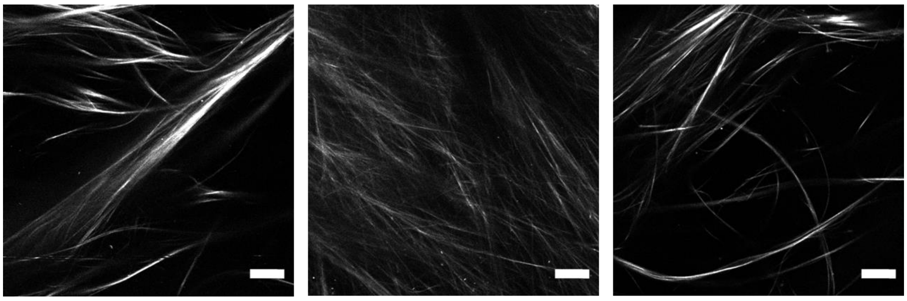

Figure S14. Further confocal images for gels of **1** prepared at a concentration of (left to right) 5 mg·mL^{-1} , 7.5 mg·mL^{-1} and 10 mg·mL^{-1} at a DMSO:water ratio of 2:8. The scale bar represents 20 μm.

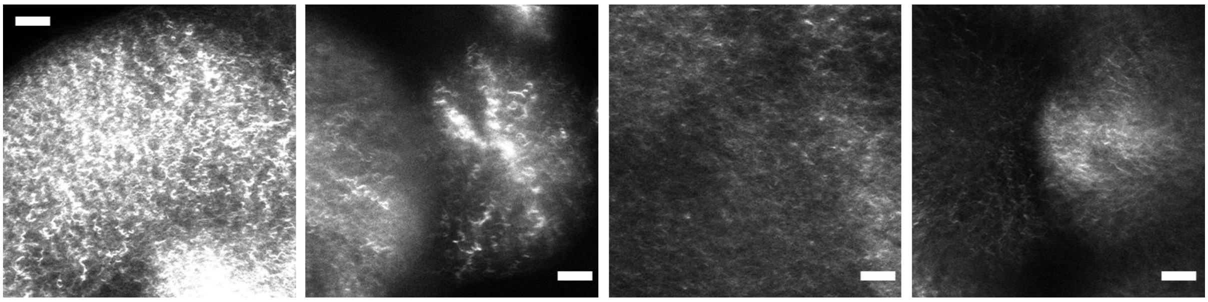

Figure S15. Further confocal images for gels of **2** prepared at a concentration of (left to right) 2.5 mg·mL^{-1} , 5 mg·mL^{-1}, 7.5 mg·mL^{-1} and 10 mg·mL^{-1} at a DMSO:water ratio of 2:8. The scale bar represents 20 μm.

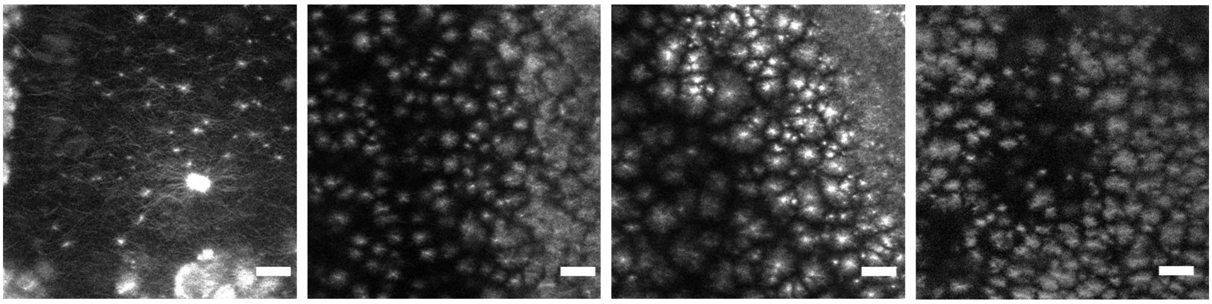

Figure S16. Further confocal images for gels of **3** prepared at a concentration of (left to right) 2.5 mg·mL^{-1} , 5 mg·mL^{-1}, 7.5 mg·mL^{-1} and 10 mg·mL^{-1} at a DMSO:water ratio of 2:8. The scale bar represents 20 μm.

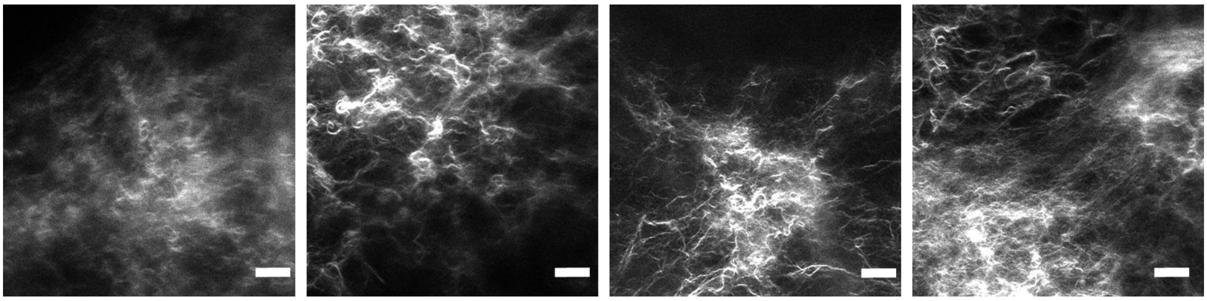

Figure S17. Further confocal images for gels of **4** prepared at a concentration of (left to right) 2.5 mg·mL^{-1} , 5 mg·mL^{-1}, 7.5 mg·mL^{-1} and 10 mg·mL^{-1} at a DMSO:water ratio of 2:8. The scale bar represents 20 μm.

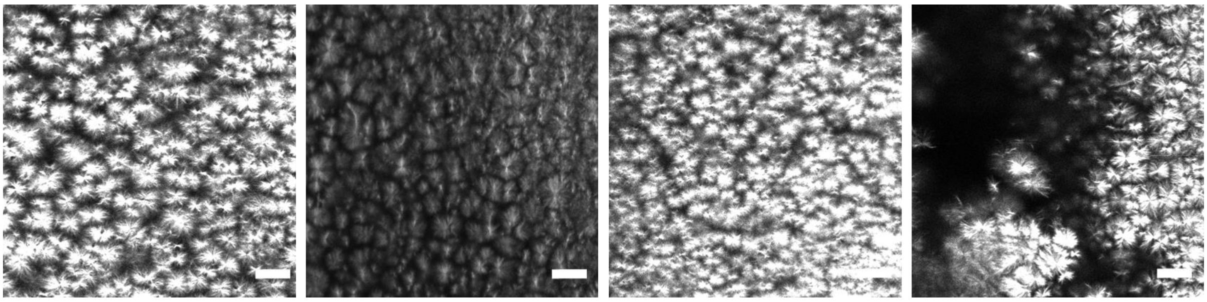

Figure S18. Further confocal images for gels of **5** prepared at a concentration of (left to right) 2.5 mg·mL^{-1} , 5 mg·mL^{-1}, 7.5 mg·mL^{-1} and 10 mg·mL^{-1} at a DMSO:water ratio of 2:8. The scale bar represents 20 μm.

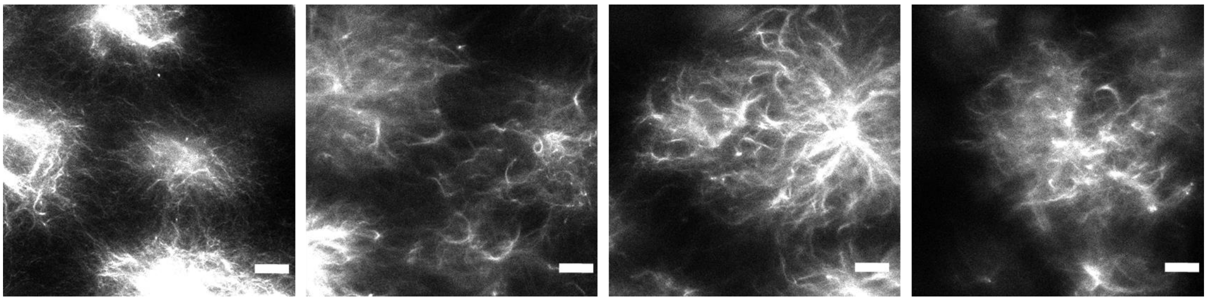

Figure S19. Further confocal images for gels of **6** prepared at a concentration of (left to right) 2.5 mg·mL^{-1} , 5 mg·mL^{-1}, 7.5 mg·mL^{-1} and 10 mg·mL^{-1} at a DMSO:water ratio of 2:8. The scale bar represents 20 μm.

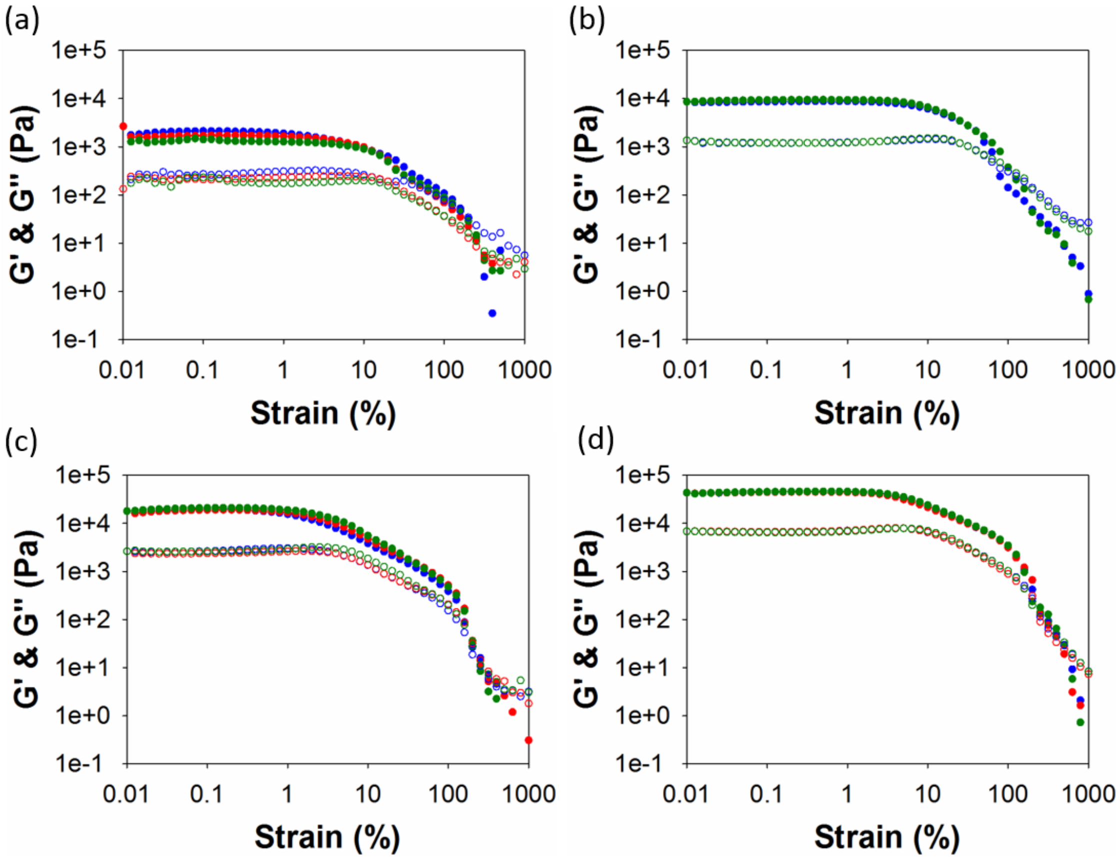

Figure S20. Strain sweeps for gels of **2** prepared at different concentrations at a DMSO:water ratio of 2:8. Concentrations are (a) 2.5 mg·mL^{-1}; (b) 5 mg·mL^{-1}; (c) 7.5 mg·mL^{-1}; (d) 10 mg·mL^{-1}. In each case, separate datasets are provided for three nominally identical gels.

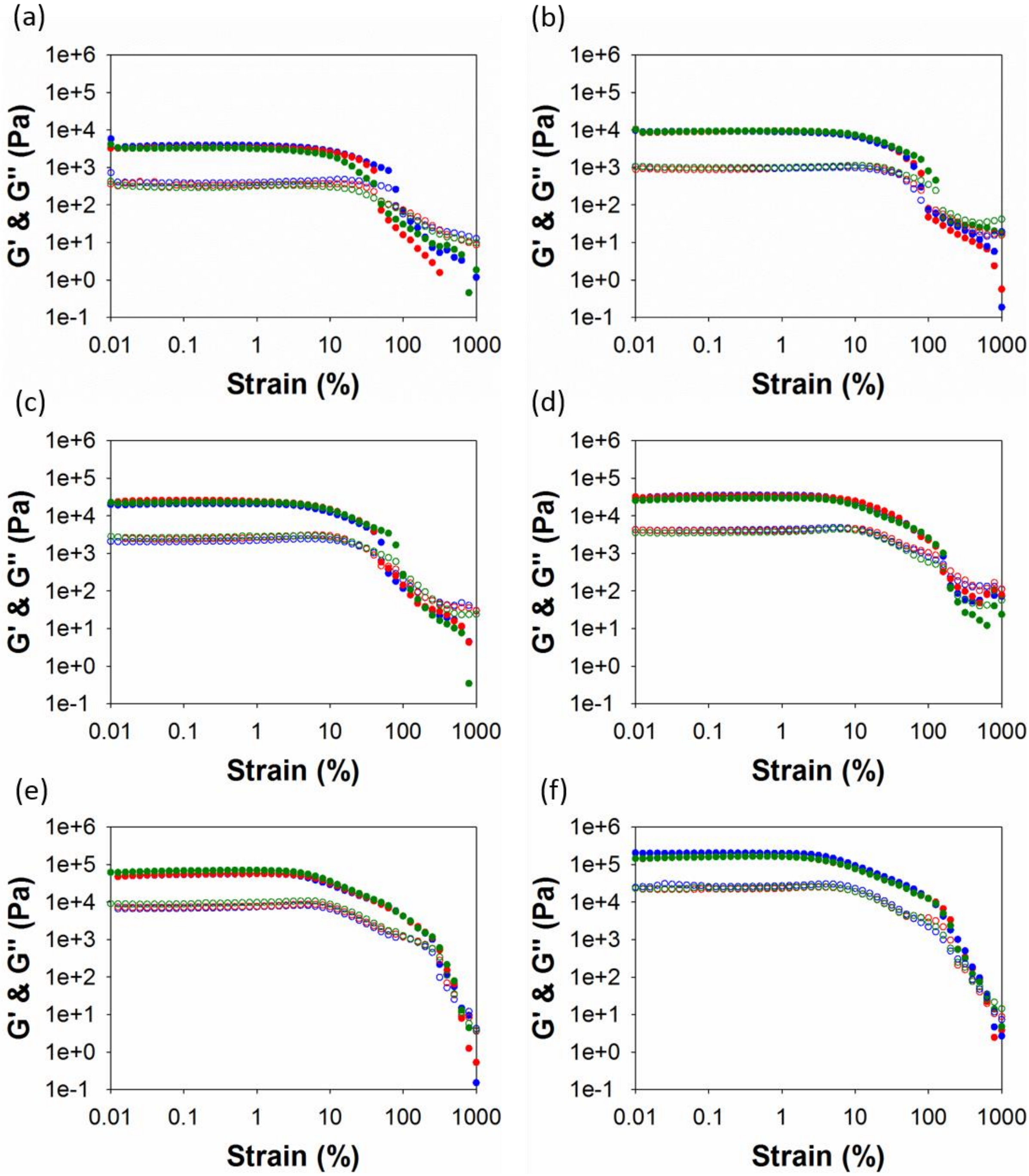

Figure S21. Strain sweeps for gels of **3** prepared at different concentrations at a DMSO:water ratio of 2:8. Concentrations are (a) 2.5 mg·mL^{-1}; (b) 3.5 mg·mL^{-1}; (c) 5 mg·mL^{-1}; (d) 7.5 mg·mL^{-1}; (e) 10 mg·mL^{-1}; (f) 20 mg·mL^{-1}. In each case, separate datasets are provided for three nominally identical gels.

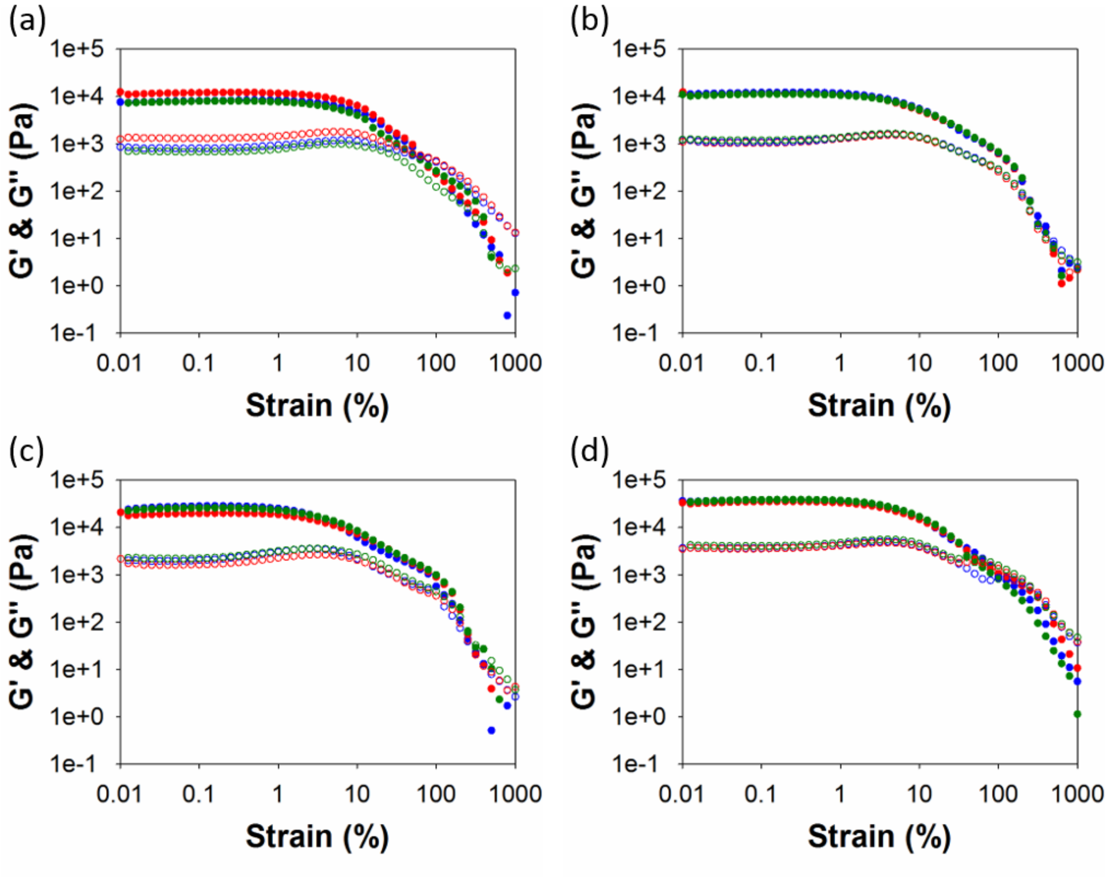

Figure S22. Strain sweeps for gels of **4** prepared at different concentrations at a DMSO:water ratio of 2:8. Concentrations are (a) 5 mg·mL^{-1}; (b) 6 mg·mL^{-1}; (c) 7.5 mg·mL^{-1}; (d) 10 mg·mL^{-1}. In each case, separate datasets are provided for three nominally identical gels.

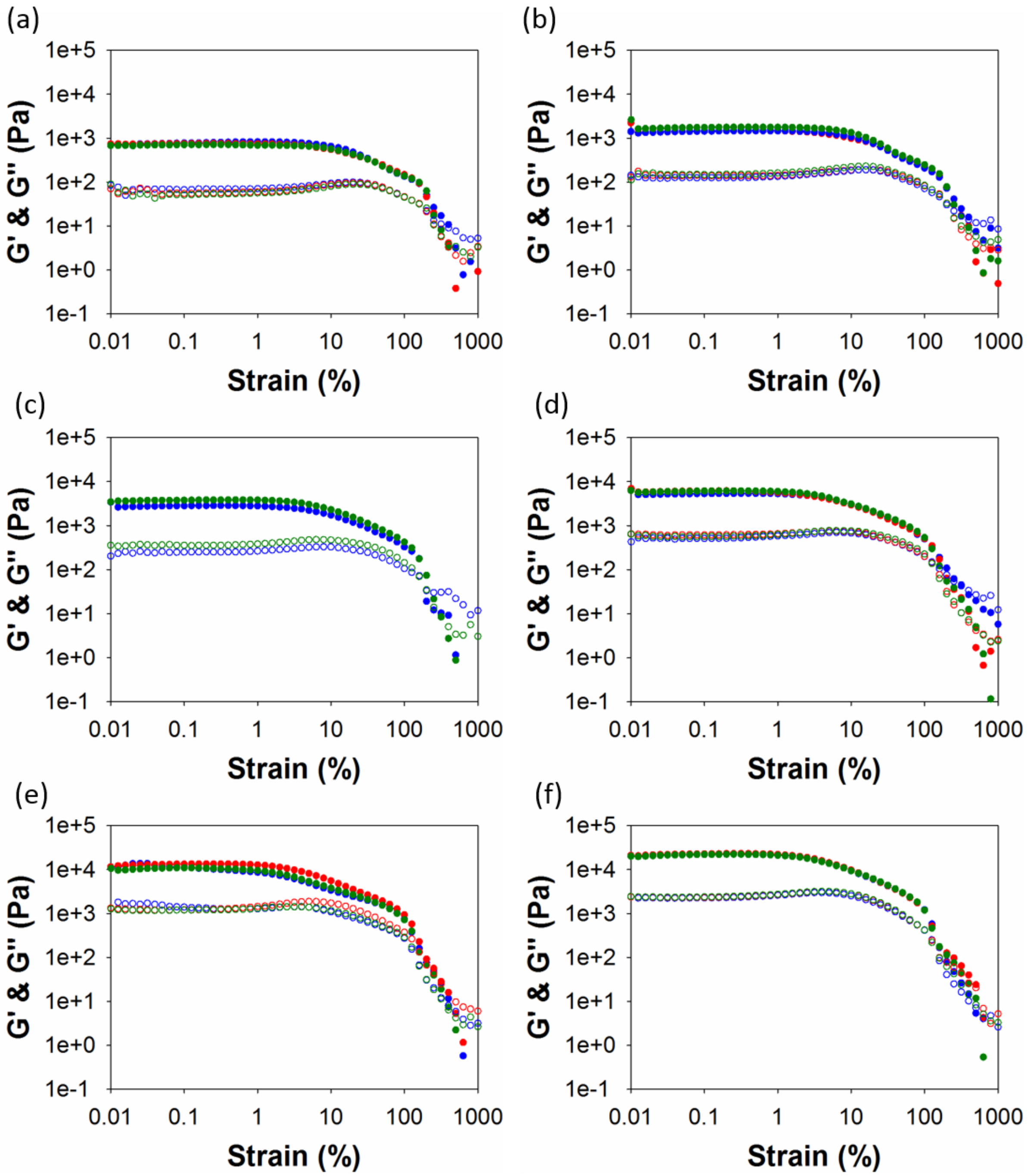

Figure S23. Strain sweeps for gels of **5** prepared at different concentrations at a DMSO:water ratio of 2:8. Concentrations are (a) 2.5 mg·mL^{-1}; (b) 3.5 mg·mL^{-1}; (c) 5 mg·mL^{-1}; (d) 6 mg·mL^{-1}; (e) 7.5 mg·mL^{-1}; (f) 10 mg·mL^{-1}. In each case, separate datasets are provided for three nominally identical gels.

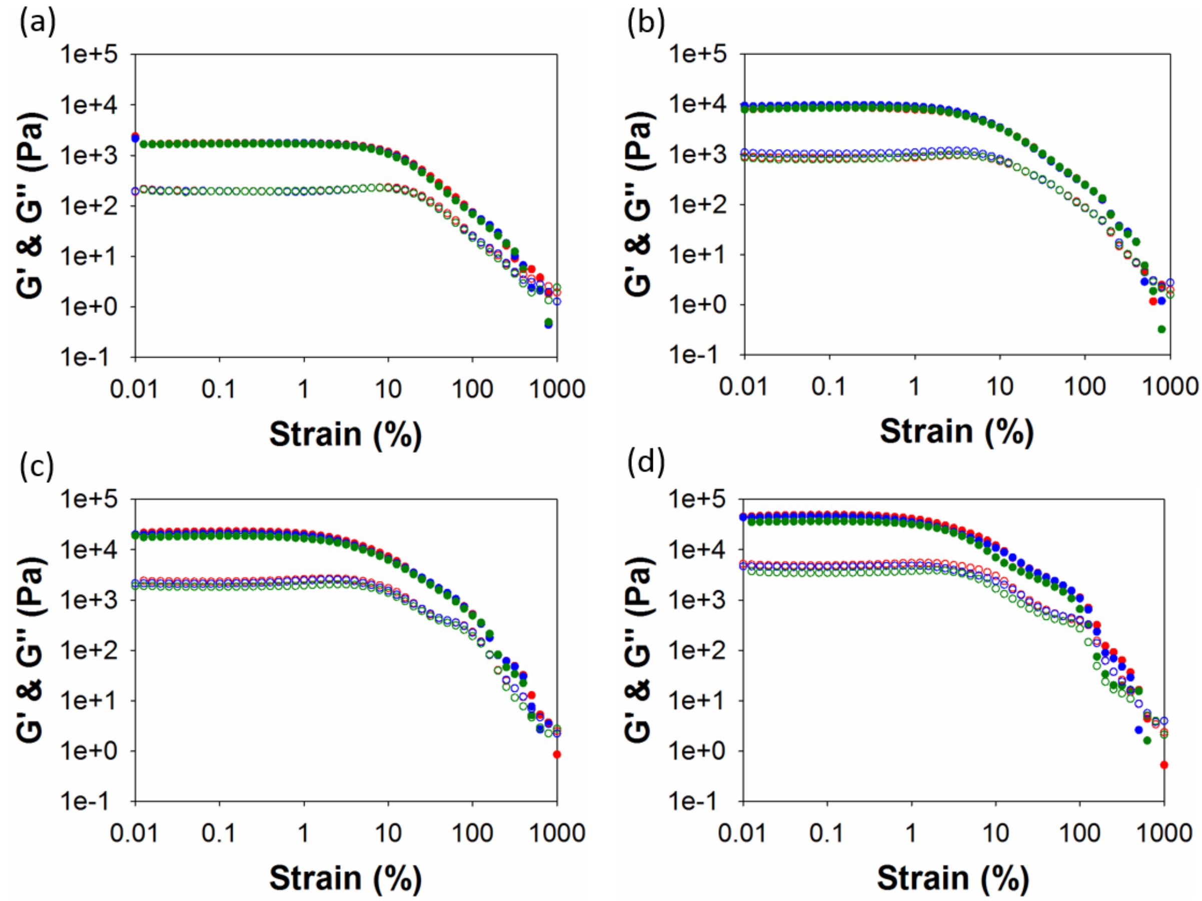

Figure S24. Strain sweeps for gels of **6** prepared at different concentrations at a DMSO:water ratio of 2:8. Concentrations are (a) 2.5 mg·mL^{-1}; (b) 5 mg·mL^{-1}; (c) 7.5 mg·mL^{-1}; (d) 10 mg·mL^{-1}. In each case, separate datasets are provided for three nominally identical gels.

Table S1. Summary of fits to the SANS data for gels **1-6**. For each gel, fitting data were collected for concentrations of 5 mg mL^{-1}, 7.5 mg mL^{-1} and 10 mg mL^{-1} (top to bottom values, respectively). Gel **1** was fitted to a power parallel model and gels **2-6** were fitted using a flexible elliptical cylinder. For gels **4**, **5** and **6**, a fixed polydispersity in radius of 0.2, 0.3 and 0.3 was chosen respectively based on quality for data fitting. All data fitted using the SasView [1] software.

	Gel 1	Gel 2	Gel 3	Gel 4	Gel 5	Gel 6
Background (cm^{-1})	344.15 E^{-5} ± 16.04 E^{-5}	269.88 E^{-5} ± 4.18 E^{-5}	276.59 E^{-5} ± 5.26 E^{-5}	392.54 E^{-5} ± 18.52 E^{-5}	582.48 E^{-5} ± 17.86 E^{-5}	506.46 E^{-5} ± 20.62 E^{-5}
	330.08 E^{-5} ± 1.38 E^{-5}	265.63 E^{-5} ± 4.20 E^{-5}	197.80 E^{-5} ± 3.93 E^{-5}	255.56 E^{-5} ± 4.02 E^{-5}	545.43 E^{-5} ± 20.36 E^{-5}	716.61 E^{-5} ± 21.06 E^{-5}
	346.76 E^{-5} ± 2.87 E^{-5}	520.09 E^{-5} ± 5.56 E^{-5}	237.37 E^{-5} ± 4.43 E^{-5}	468.96 E^{-5} ± 4.71 E^{-5}	617.46 E^{-5} ± 20.49 E^{-5}	1005.40 E^{-5} ± 21.54 E^{-5}
Scale	130.96 E^{-9} ± 3.02 E^{-9}	99.19 E^{-5} ± 0.35 E^{-5}	1952.90 E^{-6} ± 7.68 E^{-6}	994.45 E^{-6} ± 6.77 E^{-6}	427.72 E^{-6} ± 12.31 E^{-6}	977.04 E^{-6} ± 42.94 E^{-6}
	135.87 E^{-9} ± 2.34 E^{-9}	102.03 E^{-5} ± 0.35 E^{-5}	1397.10 E^{-6} ± 5.47 E^{-6}	1609.10 E^{-6} ± 6.05 E^{-6}	446.18 E^{-6} ± 14.10 E^{-6}	1398.80 E^{-6} ± 37.54 E^{-6}
	151.56 E^{-9} ± 2.36 E^{-9}	237.24 E^{-5} ± 0.57 E^{-5}	1670.20 E^{-6} ± 6.71 E^{-6}	2701.60 E^{-6} ± 11.19 E^{-6}	481.76 E^{-6} ± 13.51 E^{-6}	1882.90 E^{-6} ± 37.55 E^{-6}
Length (Å)	-	1526.10 ± 24.08	1406.30 ± 31.39	6608.60 ± 231.22	4767.10 ± 449.26	7344.90 ± 272.45
	-	1481.40 ± 21.72	1157.80 ± 19.74	3399.00 ± 112.15	12809.50 ± 446.08	8007.30 ± 189.86
	-	1749.40 ± 19.72	976.53 ± 12.02	1777.70 ± 30.91	12627.00 ± 396.94	8230.50 ± 150.61
Kuhn Length (Å)	-	239.44 ± 6.13	332.31 ± 4.48	211.42 ± 5.06	258.57 ± 30.96	79.07 ± 4.24
	-	223.06 ± 5.63	390.08 ± 4.67	185.69 ± 7.46	70.01 ± 2.84	77.34 ± 2.52
	-	241.80 ± 3.29	244.11 ± 1.54 E^{-3}	444.39 ± 3.30 E^{-3}	69.63 ± 2.52	77.70 ± 1.88
Radius (Å)	-	30.18 ± 0.07	34.13 ± 0.06	41.57 ± 0.13	48.22 ± 0.49	40.84 ± 0.61
	-	30.85 ± 0.08	34.81 ± 0.07	52.68 ± 0.23	36.71 ± 0.30	41.00 ± 0.35
	-	28.53 ± 0.04	35.38 ± 0.07	63.98 ± 0.06	36.33 ± 0.26	41.12 ± 0.25
Axis ratio	-	2.74 ± 0.02	1.78 ± 0.01	3.25 ± 0.03	3.03 ± 0.14	1.99 ± 0.03
	-	2.70 ± 0.02	1.86 ± 0.01	2.44 ± 0.03	2.91 ± 0.03	2.14 ± 0.02
	-	2.80 ± 0.01	1.71 ± 0.01	2.75 ± 0.01	3.14 ± 0.03	2.27 ± 0.02
Power	3.65 ± 4.65 E^{-3}	-	-	-	-	-
	3.91 ± 3.76 E^{-3}	-	-	-	-	-
	3.92 ± 3.41 E^{-3}	-	-	-	-	-
Polydispersity in radius	-	-	0.1	0.2	0.3	0.3
	-	-	-	0.2	0.3	0.3
	-	-	-	0.2	0.3	0.3
χ^2	5.61	1.31	3.51	3.47	1.64	1.72
	2.97	1.35	1.87	2.64	2.42	1.97
	3.06	4.97	2.18	3.71	3.39	3.48

References

1. SasView. Available onlie: www.sasview.org (accessed on 24th October 2018).
